# Registration uncertainties between 3D cone beam computed tomography and different reference CT datasets in lung stereotactic body radiation therapy

**DOI:** 10.1186/s13014-016-0720-9

**Published:** 2016-10-26

**Authors:** Markus Oechsner, Barbara Chizzali, Michal Devecka, Stephanie Elisabeth Combs, Jan Jakob Wilkens, Marciana Nona Duma

**Affiliations:** 1 Department of Radiation Oncology, Klinikum rechts der Isar, Technical University of Munich, Ismaninger Str. 22, 81675 Munich, Germany; 2 Institute of Innovative Radiotherapy (iRT), Helmholtz Zentrum München, Ingolstädter Landstraße 1, Munich, Germany

**Keywords:** SBRT, Average intensity projection, Maximum intensity projection, Mid-ventilation, CBCT, Image registration

## Abstract

**Background:**

The aim of this study was to analyze differences in couch shifts (setup errors) resulting from image registration of different CT datasets with free breathing cone beam CTs (FB-CBCT). As well automatic as manual image registrations were performed and registration results were correlated to tumor characteristics.

**Methods:**

FB-CBCT image registration was performed for 49 patients with lung lesions using slow planning CT (PCT), average intensity projection (AIP), maximum intensity projection (MIP) and mid-ventilation CTs (MidV) as reference images. Both, automatic and manual image registrations were applied. Shift differences were evaluated between the registered CT datasets for automatic and manual registration, respectively. Furthermore, differences between automatic and manual registration were analyzed for the same CT datasets. The registration results were statistically analyzed and correlated to tumor characteristics (3D tumor motion, tumor volume, superior-inferior (SI) distance, tumor environment).

**Results:**

Median 3D shift differences over all patients were between 0.5 mm (AIPvsMIP) and 1.9 mm (MIPvsPCT and MidVvsPCT) for the automatic registration and between 1.8 mm (AIPvsPCT) and 2.8 mm (MIPvsPCT and MidVvsPCT) for the manual registration. For some patients, large shift differences (>5.0 mm) were found (maximum 10.5 mm, automatic registration).

Comparing automatic vs manual registrations for the same reference CTs, ∆AIP achieved the smallest (1.1 mm) and ∆MIP the largest (1.9 mm) median 3D shift differences. The standard deviation (variability) for the 3D shift differences was also the smallest for ∆AIP (1.1 mm). Significant correlations (*p* < 0.01) between 3D shift difference and 3D tumor motion (AIPvsMIP, MIPvsMidV) and SI distance (AIPvsMIP) (automatic) and also for 3D tumor motion (∆PCT, ∆MidV; automatic vs manual) were found.

**Conclusions:**

Using different CT datasets for image registration with FB-CBCTs can result in different 3D couch shifts. Manual registrations achieved partly different 3D shifts than automatic registrations. AIP CTs yielded the smallest shift differences and might be the most appropriate CT dataset for registration with 3D FB-CBCTs.

## Introduction

Nowadays, several CT datasets are available for the planning process in stereotactic body radiation therapy (SBRT). A slow CT scan for treatment planning (PCT) can be acquired [1, 2]. Respiratory-correlated four-dimensional CT (4DCT) is the standard in SBRT to characterize tumor motion and to reduce respiratory-induced image artifacts [3, 4]. From the 4DCT further 3D datasets can be generated. The maximum intensity projection (MIP) CT shows the maximum intensity of each voxel over all phases, whereas the average intensity projection (AIP) CT represents the mean intensity of each voxel over the breathing cycle. A mid-ventilation (MidV) CT can be selected as the 4DCT phase showing the tumor in its near-mean position during the breathing cycle [5].

Several works exist comparing PCT, MIP or AIP CT datasets for delineation of moving targets [6–10]. Furthermore, dose differences for the planning target volume (PTV) and organs at risk (OARs) resulting from using different CT datasets for dose calculation in lung SBRT were evaluated [11–13]. Usually, the CT dataset used for treatment planning is also used as reference CT dataset for alignment with the free breathing cone beam CT (FB-CBCT) images which are acquired for image guided radiation therapy (IGRT). Using kilovoltage 3D FB-CBCT for patient positioning was proofed to increase positioning accuracy and enables a reduction of safety margins [14–18]. Nowadays, FB-CBCTs are the most common 3D imaging modality for IGRT. Recently, respiratory correlated CBCT (4D-CBCT) has been commercialized. 4D-CBCT acquisition is more time consuming than using FB-CBCT and different results were published if a 4D-CBCT workflow improves patient positioning in comparison to FB-CBCT [19–23].

Up to now, only few studies investigated differences in patient positioning depending on the applied reference CT dataset for image registration with FB-CBCT [24, 25], by comparing only two CT datasets against each other. Furthermore, in these studies image registration was performed solely using automatic registration tools. In clinical practice however, the automatic registration results are reviewed by radiation oncologists and/or radiotherapy technicians who may perform manual corrections due to their visual judgement.

The aim of this study was therefore to evaluate differences in couch shifts resulting from using four different CT datasets (PCT, AIP, MIP, MidV) as reference for image registration with FB- CBCTs. We compared couch shifts yielded by automatic and manual image registration as well as the impact of different tumor characteristics on the registration results.

## Material and methods

### Patients and image acquisition

Forty-nine lung SBRT patients which were treated in our department were retrospectively selected for this study. The tumor characteristics of these patients are listed in Table 1. All patients underwent CT imaging for treatment planning whereas they were immobilized using a vacuum couch and low pressure foil (Medical Intelligence GmbH, Schwabmünchen, Germany). Additionally, the patients received oxygen supply to further reduce respiratory motion. First, a slow 3D-CT scan (PCT) was acquired (slice thickness 3.0 mm, in plane resolution 1.0 × 1.0 mm). Afterwards, a 4DCT scan was performed. The respiratory position management system (RPM, Varian Medical Systems, Palo Alto, CA, USA) was used to monitor the breathing motion of the patients by tracking an infrared marker positioned at the thorax. Using these motion information, the 4DCT was binned into 10 phases covering the whole breathing cycle (slice thickness 2.1 mm, in plane resolution 1.0 × 1.0 mm). From these 10 phases an AIP and a MIP CT dataset were calculated using the Eclipse 13.0 software (Varian Medical Systems, Palo Alto, CA, USA). To select the mid-ventilation CT for each patient, the tumor was delineated as gross target volume (GTV) in all 10 phases of the corresponding 4DCT. The tumor motion was determined by evaluating the center of mass (COM) coordinates of the gross tumor volumes (GTVs) in all phases. The time-weighted mean tumor position was determined and the phase which was closest to this mean tumor position was selected as MidV [5]. All patients were treated on a Varian Clinac Trilogy linear accelerator, equipped with an on board kilovoltage imaging system. The same patient immobilization was used during treatment as for CT scanning. Before treatment, FB-CBCTs were acquired for IGRT. In order to reduce the complexity of the study, only the FB-CBCTs of the first day of treatment were used for image registration with the corresponding PCT, AIP, MIP and MidV CTs of the 49 patients.

**Table 1:** Patients and tumor characteristics

	Median (range)^*^ or cases^+^	
Number of patients		49 ^+^
Tumor volume [cm^3^]		8.9 (0.6–119.0)^*^
3D tumor motion [cm]		0.7 (0.1–2.6)^*^
Tumor location	upper LL, left	12 ^+^
	lower LL, left	9 ^+^
	upper LL, right	6 ^+^
	middle LL, right	14 ^+^
	lower LL, right	8 ^+^
3D tumor motion [cm]	<0.5	16 ^+^
	0.5–1.0	20 ^+^
	1.0–1.5	6 ^+^
	>1.5	7 ^+^
Tumor volume [cm^3^]	<10	28 ^+^
	10–20	10 ^+^
	>20	11 ^+^
SI distance [cm]	<0.0	9 ^+^
	0.0–5.0	34 ^+^
	>5.0	6 ^+^
Tumor environment	adherent	14 ^+^
	adjacent	7 ^+^
	free	28 ^+^

Patients and tumor characteristics

*LL* lung lobe; *SI* superior-inferior; ^*^ denotes measured values; ^+^ denotes number of cases

### Image registration

Image registration of the CT datasets was performed with the image registration software implemented in Eclipse 13.0. The FB-CBCT of each patient was registered to the corresponding PCT, AIP, MIP and MidV CT dataset. The registration aim was to achieve the best alignment between the visible tumor in the FB-CBCT and the reference CT. Only 3D translational shifts in left-right (x), anterior-posterior (y) and superior-inferior (z) direction were used.

Two kinds of image registration were performed: an automatic and a manual image registration. For the automatic image registration, a coarse manual registration was performed beforehand to roughly align the CT datasets. Subsequently, a volume of interest was placed in the FB-CBCT around the visible tumor to restrict the registration to this area. Then the automatic image registration was performed while the optimizer (“Downhill Simplex”) was performing a similarity measure using mutual information. Finally, the result of the automatic registration was visually reviewed to exclude obviously inaccurate registrations.

The manual image registration was always carried out by the same radiation oncologist. An automatic image registration was initially executed; afterwards, correction shifts were manually applied to align the tumor volume represented in the FB-CBCT to the tumor volume represented in the CT datasets. Additionally, the contour of the internal target volume (ITV) could be blended in the registered images as assistance. For the ITV generation, GTV volumes were contoured in all phases of the 4DCT and merged together. The best manual registration was defined due to the visual judgement of the radiation oncologist.

### Data analysis and statistics

The x, y and z coordinates of the registered images were evaluated. For the automatic and manual registration, the differences between the coordinates of the registered CT datasets were calculated. Additionally, a 3D shift vector was calculated from the differences of the x, y and z coordinates. Furthermore, 3D shift vectors were also calculated for coordinate differences between the automatic and manual registration for the same CT datasets.

Two main registration issues will be analyzed. First, registration differences which arise if different reference CT datasets are used for image registration with FB-CBCTs. This issue was analyzed for the automatic and manual registration, respectively. The comparison of coordinate differences between registrations with different reference CT datasets will be denoted in accordance with the pattern CTx vs CTy (e.g. AIPvsPCT for the coordinate difference between AIP to FB-CBCT registration and PCT to FB-CBCT registration).

Second, the registration difference between the automatic and manual registration if the same reference CT dataset is used for the registration with FB-CBCT. The comparison of coordinate differences between automatic and manual registration for the same CT dataset will be denoted as ∆CTx (e.g. ∆PCT: comparing coordinates of PCT to FB-CBCT registration (automatic) with PCT to FB-CBCT registration (manual)).

In addition, several tumor characteristics were determined (tumor motion, volume, location and tumor environment) and correlated to the registration differences. Tumor motion was calculated as 3D motion vector from the COM coordinates of the GTVs, contoured in all 10 phases of the 4DCTs. Two groups were made for further statistical analysis: GTVs with <5 mm 3D motion and >5 mm 3D motion. The tumor volume was the volume of the GTV contoured in the PCT. The location of the tumor was evaluated as the distance between the CT slice where the tracheal carina was visible and the GTV in superior-inferior direction. GTVs located cranial to tracheal carina were measured as negative and GTVs caudal were measured as positive distances. Additionally, we tested if the environment of the tumor influences the registration results. For that purpose, the tumor environment was classified into three groups according to the distance to dense tissue (e.g. diaphragm, chest wall, mediastinum). Tumors in the first group have clear contact to dense tissue (=adherent). The second group consists of tumors having small distances (≤5 mm) to dense tissue (=adjacent). The third group included all tumors with distances >5 mm (=free).

Statistical evaluation was performed using SPSS 23.0 (SPSS Inc, Chicago, IL, USA). The Wilcoxon test (two-sided) was used to compare the registration results of the different CT datasets. The Mann-Whitney test was performed to test for significant differences between patient groups with 3D tumor motion <5 mm and >5 mm. A p-value <0.05 was considered to indicate statistical significance. Correlations between registration results and tumor characteristics were analyzed using Spearman’s rank correlation (R_s_).

## Results

**Fig. 1:**
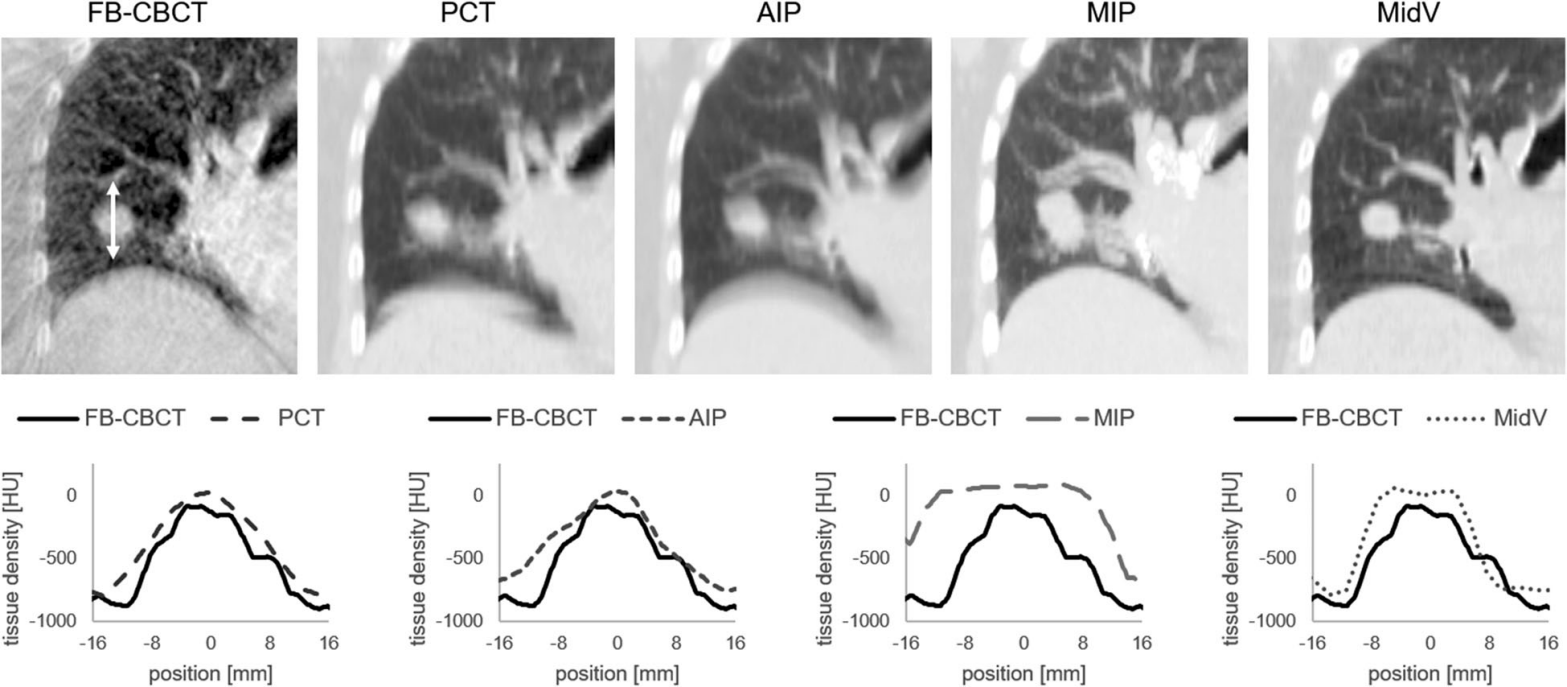
Tumor depiction in the different CT datasets. The visibility of the tumor of a lung patient in the CT datasets is presented in the upper part. Line profiles (lower part) show the density variation of the tumor in z-direction (superior-inferior) in the CT datasets in comparison to FB-CBCT. FB-CBCT = free breathing cone beam CT; PCT = slow planning CT; AIP = average intensity projection; MIP = maximum intensity projection; MidV = mid-ventilation

Figure 1 shows an example of the visibility of the tumor in the different CT datasets. In FB-CBCT, PCT and AIP the tumor seems blurred due to motion. MIP depicts the envelope of the moving tumor with the maximum pixel density during the breathing cycle whereas MidV is a snapshot of the tumor during a short phase in the breathing cycle.

The median, minimal and maximal registration differences in x, y and z direction between the registered CT datasets over all patients are depicted in Table 2. Median differences over all patients were small between the CT registrations (≤ ± 0.6 mm) whereas the range of shifts showed large differences. These were mostly pronounced in z-direction (superior-inferior) which is the direction of the largest tumor motion. Wilcoxon test yielded some statistically significant differences (*p* < 0.05, Table 2), mostly to MIP registrations. Two patients showed large differences in the automatic registration (max. 3D shift up to 9.9 mm and 10.5 mm). Both patients had small tumor volumes (≤2.3 cm^3^) and large tumor motion (1.6 cm). The manual registration resulted in distinctly smaller registration differences for these patients (3D shift ≤6.4 mm).

**Table 2:** Registration differences between the CT datasets

	Δ couch shift; median (minimum/maximum)					
Registration	Δx [mm]	*p* value	Δy [mm]	*p* value	Δz [mm]	*p* value
Automatic						
AIPvsPCT	−0.5 (−2.1/2.7)	<0.01*	−0.1 (−2.9/2.2)	0.40	−0.1 (−8.5/3.2)	0.16
MIPvsPCT	−0.5 (−1.3/2.9)	<0.01*	−0.4 (−4.0/2.3)	0.03*	−0.3 (−8.7/3.3)	0.03*
MidVvsPCT	−0.3 (2.0/2.2)	0.02*	0.0 (−2.9/3.9)	0.86	−0.4 (−9.9/5.8)	0.05
AIPvsMIP	−0.1 (−1.0/1.2)	0.13	0.2 (−0.8/1.5)	<0.01*	0.1 (−3.0/4.3)	0.06
AIPvsMidV	−0.1 (−3.8/1.6)	0.29	−0.2 (−2.8/2.3)	0.16	0.2 (−6.3/2.7)	0.11
MIPvsMidV	−0.1 (−2.8/1.6)	0.65	−0.5 (−2.8/2.2)	<0.01*	0.0 (−9.9/5.7)	0.89
Manual						
AIPvsPCT	0.1 (−2.3/3.5)	0.08	0.1 (−3.1/3.5)	0.68	0.4 (−3.9/4.9)	0.32
MIPvsPCT	0.5 (−4.5/3.3)	0.04*	−0.6 (−4.3/4.8)	0.27	0.6 (−4.3/4.0)	0.05
MidVvsPCT	0.2 (−3.5/4.3)	0.12	−0.2 (−4.3/3.9)	0.23	0.5 (−4.6/5.6)	0.19
AIPvsMIP	0.0 (−2.3/3.5)	0.61	0.2 (−2.6/4.1)	0.12	−0.3 (−6.5/3.2)	0.46
AIPvsMidV	0.0 (−2.3/2.2)	0.70	0.1 (−2.6/3.6)	0.19	−0.1 (−3.5/5.6)	0.63
MIPvsMidV	0.0 (−3.8/3.1)	0.71	0.0 (−4.2/4.2)	0.85	0.1 (−4.7/5.4)	0.81
Automatic vs manual						
ΔPCT	−0.2 (−3.1/3.1)	0.22	0.0 (−4.4/2.7)	0.82	0.0 (−8.1/4.0)	0.90
ΔAIP	0.0 (−3.5/2.5)	0.55	−0.2 (−1.5/2.0)	0.41	−0.1 (−6.0/3.0)	0.99
ΔMIP	−0.2 (−2.8/3.9)	0.13	0.5 (−1.7/4.6)	0.01*	−0.1 (−3.9/4.4)	0.90
ΔMidV	−0.2 (−3.7/1.7)	0.30	0.1 (−4.4/6.4)	0.90	0.2 (−9.9/2.9)	0.24

Median, minimal and maximal differences of couch shifts between the registered CT datasets in x, y and z direction over all patients

*PCT* slow planning CT, *AIP* average intensity projection; *MIP* maximum intensity projection; *MidV* mid-ventilation

*indicates statistically significant differences *p* < 0.05

**Fig. 2:**
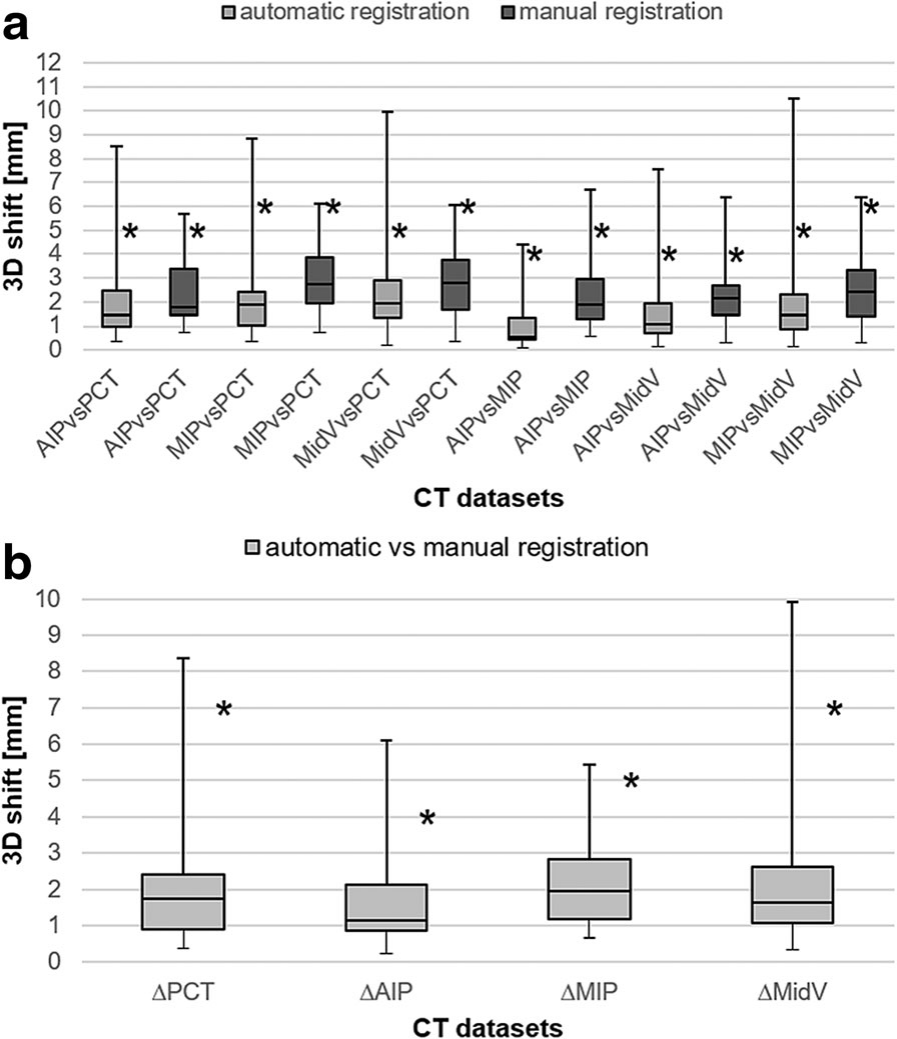
Box-whisker-plots of the 3D shift differences. Box-whisker-plots show the 3D shift differences over all patients for the automatic and manual registration between the CT datasets (**a**) and between the automatic and manual registration for the same CT dataset (**b**). The boxplots represent the 25 % quartile, median and 75 % quartile. Whiskers mark the minimal and maximal differences. Asterisks represent the 3D shift threshold including 95 % of the patients. PCT = slow planning CT; AIP = average intensity projection; MIP = maximum intensity projection; MidV = mid-ventilation

The differences of the 3D shift vector between the registered CT datasets are depicted as boxplots in Fig. 2. Median differences were always larger for the manual registrations than for the automatic registration with a mean difference of 0.9 mm between automatic and manual registration. Otherwise the maximum differences between the registered CT datasets were larger for the automatic registration (≤10.5 mm) than for the manual registration (≤6.7 mm). Automatic registration yielded the smallest median difference between AIPvsMIP (3D shift = 0.5 mm). The manual registration achieved the best median agreements between AIPvsMIP and AIPvsPCT (3D shift = 1.9 mm and 1.8 mm). Comparing the difference between automatic and manual registration for the same CT dataset, ∆AIP achieved the best median agreement (3D shift = 1.1 mm) whereas the largest differences were found for ∆MIP (3D shift = 1.9 mm).

The variability of the 3D shift differences is expressed as one standard deviation (SD) and is depicted in Table 3, together with the 3D shift range. The automatic registration achieved the smallest SD for AIPvsMIP (0.9 mm) and the manual registration for AIPvsMidV (1.1 mm). In total, the variabilities between registrations with different reference CT datasets were slightly smaller for the manual registration than for the automatic registration. Comparing the variability between automatic and manual registration for the same CT datasets, ∆AIP resulted in the smallest value (1.1 mm). The largest SD was found for ∆MidV (1.9 mm).

**Table 3:** 3D registration differences

Δ3D couch shift; SD and range (minimum/maximum)							
	Automatic		Manual			Automatic vs manual	
	SD [mm]	Range [mm]	SD [mm]	Range [mm]		SD [mm]	Range [mm]
AIPvsPCT	1.5	0.3/8.5	1.3	0.8/5.7	∆PCT	1.7	0.4/8.3
MIPvsPCT	1.7	0.4/8.8	1.4	0.7/6.1	∆AIP	1.1	0.2/6.1
MidVvsPCT	1.5	0.2/9.9	1.5	0.4/6.1	∆MIP	1.2	0.7/5.4
AIPvsMIP	0.9	0.1/4.4	1.2	0.6/6.7	∆MidV	1.9	0.3/9.9
AIPvsMidV	1.3	0.1/7.6	1.1	0.3/6.4			
MIPvsMidV	1.8	0.1/10.5	1.6	0.3/6.4			

SD and range (minimum and maximum) of 3D couch shift differences between the registered CT datasets over all patients

*PCT* slow planning CT, *AIP* average intensity projection; *MIP* maximum intensity projection; *MidV* mid-ventilation

Overall the median differences were not large between the registrations, nevertheless if we aim to encompass 95 % of the data of the patients (marked with asterisk in Fig. 2), the registration uncertainties could be as high as 6 mm with the smallest thresholds (4 mm) for AIPvsMIP and AIPvsMidV (automatic) and AIPvsMidV (manual). Even if comparing differences between the same CT datasets, the smallest 3D shift thresholds (4 mm) were found for ∆AIP if 95 % of the data of the patients are assessed.

Spearman’s correlation coefficients were calculated between 3D shifts and tumor characteristics. All correlations with statistical significance (*p* < 0.05) are listed in Table 4. Most registration differences showed only weak correlations (*R*_s_ < ±0.5) to tumor characteristics with some exceptions. Tumor motion achieved a good correlation to 3D shift differences between AIPvsMIP (*R*_s_ = 0.733) or MIPvsMidV (*R*_s_ = 0.686) for the automatic registration just as SI distance to 3D shift difference between AIPvsMIP (*R*_s_ = 0.505). The differences between automatic vs manual registration for ∆PCT and ∆MidV correlated also with tumor motion (*R*_s_ = 0.635 and 0.568). Less and weaker correlations were found for manual than for automatic registrations. Tumor volume and tumor environment did not correlate to shift differences between automatic vs manual registrations.

**Table 4:** Spearman’s correlation coefficient Rs

Tumor characteristics	Automatic		Manual		Automatic vs manual	
	CTs	R_s_	CTs	R_s_	CTs	R_s_
Tumor motion	MIPvsPCT	0.482				
	MidVvsPCT	0.353			∆PCT	0.635*
	AIPvsMIP	0.733*	MIPvsPCT	0.288	∆AIP	0.430
	AIPvsMidV	0.429	MIPvsMidV	0.488	∆MidV	0.568*
	MIPvsMidV	0.686*				
Tumor volume	AIPvsMidV	−0.310	MIPvsPCT	0.324	–	–
SI distance	MIPvsPCT	0.286	^_^	–	∆MidV	0.286
	AIPvsMIP	0.505*				
	MIPvsMidV	0.355				
Tumor environment	AIPvsMidV	−0.338	MIPvsPCT	0.294	–	–

Spearman’s correlation coefficient Rs between tumor characteristics and the 3D shift vector. Presented are registrations reaching significant correlations (*p* < 0.05)

*PCT* slow planning CT, *AIP* average intensity projection; *MIP* maximum intensity projection; *MidV* mid-ventilation

*indicates statistically significant differences with *p* < 0.01

**Fig. 3:**
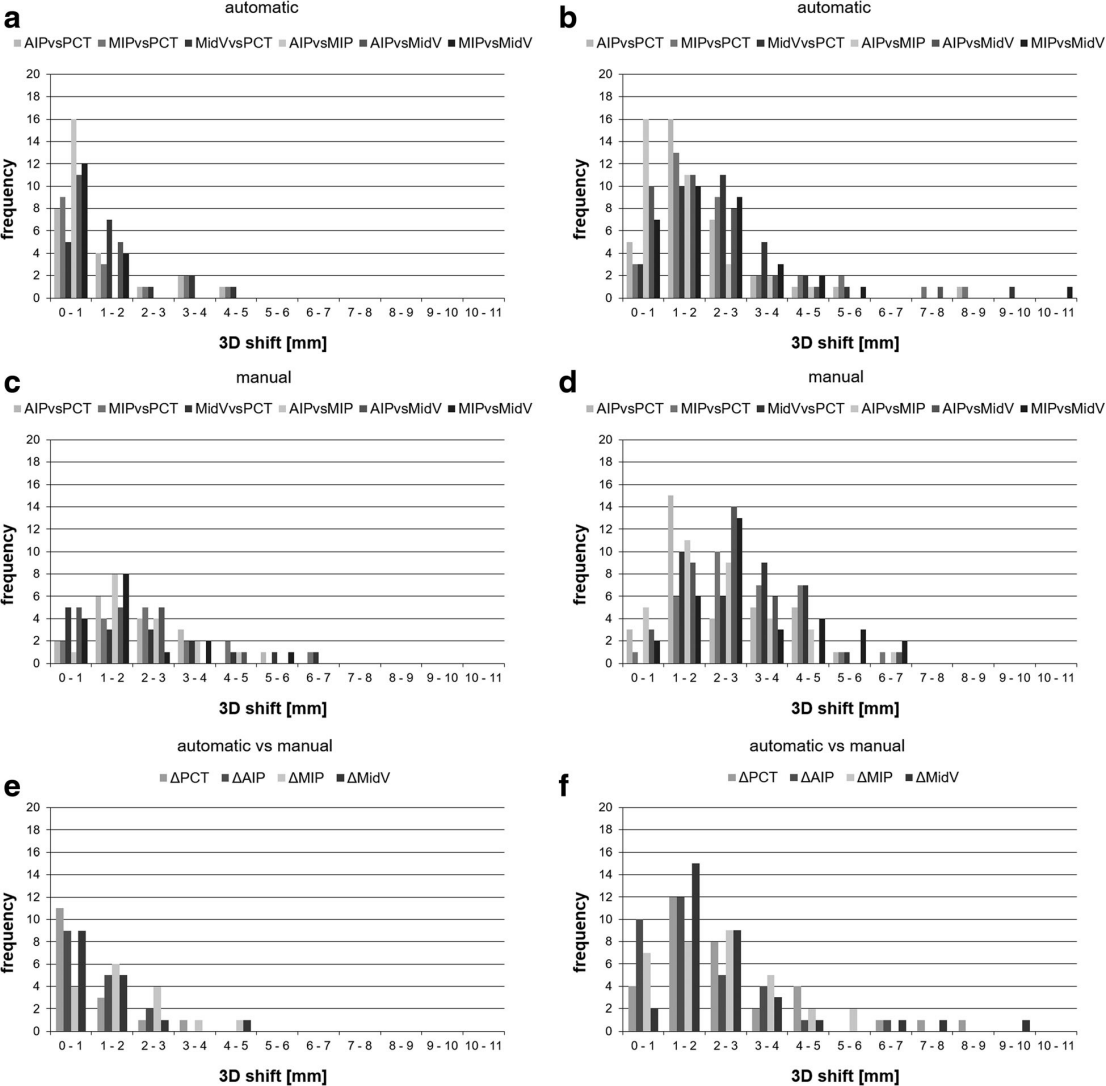
Frequencies of 3D shift differences between the CT datasets. 3D shifts are sorted at intervals of 1.0 mm and are presented for automatic, manual and automatic vs manual registrations. The barplots left (**a**, **c**, **e**) show the 3D shift frequencies for patients with tumor 3D motion <5 mm (16 patients), the right side (**b**, **d**, **f**) with tumor 3D motion >5 mm (33 patients). PCT = slow planning CT; AIP = average intensity projection; MIP = maximum intensity projection; MidV = mid-ventilation

Frequencies of 3D shift differences between the registered CT datasets for patient groups with 3D tumor motion <5 mm and >5 mm are depicted in Fig. 3. Overall, six patients had at least one 3D shift difference larger than 5 mm for the automatic registration and nine patients for the manual registration. For the automatic vs manual registration, seven patients had differences larger than 5 mm.

The mean 3D shift differences between the patient groups with 3D tumor motion <5 mm and >5 mm were significantly different (*p* < 0.05) for all automatic registrations except for MidVvsPCT (*p* = 0.06). The mean differences ranged between 0.6 mm (AIPvsPCT) and 1.6 mm (MIPvsMidV). When assessing manual registration, only AIPvsMidV and MIPvsMidV reached statistically significance with changes in mean 3D shift differences from 0.1 mm (AIPvsPCT) up to 1.3 mm (MIPvsMidV). Automatic vs manual registration resulted in significant mean 3D shift differences between both groups for ∆PCT, ∆AIP and ∆MidV, but not for ∆MIP. The smallest mean changes were 0.4 mm (∆MIP) and the largest 1.5 mm (∆MidV).

## Discussion

The results of this study show, that the choice of the reference CT dataset for image registration with FB-CBCTs considerably affects the registration results for patient positioning. Our study compared registrations of four different CT datasets for a large sample of 49 lung SBRT patients. These four CT datasets were already applied for contouring [6–10] and treatment planning [5, 11–13] in previous works. Further, automatic and manual image registration was applied resulting in differences between both registration types.

Guidelines for volumetric IGRT are available [26, 27] but no recommendations are given whether image registration should be performed manually or automatically. In our clinical practice, manual image registration between FB-CBCT and a reference CT is routinely performed in lung SBRT. Initially automatic registration is applied and the result is subsequently reviewed by a radiation oncologist who adds manual corrections depending on his visual assessment. The first aim of our study was to assess registration differences between different CT datasets.

Overall, the automatic registration achieved smaller median 3D shift differences than the manual registration. Automatic registration yielded the smallest 3D shift for AIPvsMIP (0.5 mm) and manual registration for AIPvsMIP and AIPvsPCT (1.9 mm and 1.8 mm). Otherwise, variabilities (SD) were higher for the automatic than for the manual registration, except for AIPvsMIP. In a few cases automatic registration resulted in large shift differences between registration with different CT datasets (up to 10.5 mm).

It is difficult however to state what the “right” registration is, which is a general issue for treatments of moving targets in clinical practice. The automatic registration uses the density information of the CT datasets. Registration results depend on the applied algorithm but are not subjective, in contrast to manual registration. Nonetheless visual assessment is still a “gold standard” in clinical image registration.

In the literature there are two studies on patients available, both performing solely automatic registration. Shirai et al. [25] compared AIP and MIP registrations to FB-CBCTs for 16 patients with isolated lung lesions (64 FB-CBCTs in total). They found a significant shift in inferior direction (z) (mean ± SD: -0.6 ± 1.0 mm) after MIP registration compared to AIP registration. Jiang et al. [24] compared setup errors obtained with FB-CBCTs registered to free breathing 3D-CT and AIP (14 lung cancer patients, 142 FB-CBCTs in total). Significantly larger setup errors in all directions were reported for free breathing 3D-CT. Both studies concluded that AIP should be the preferred reference image set whereby only two CT datasets were compared against each other.

Several groups performed studies using moving phantoms to investigate and compare FB-CBCT to CT imaging under reference conditions [23–25, 28–30]. Registration differences using AIP and MIP were investigated in Ref. [25]. Larger registration errors were found for MIP than for AIP and the authors stated that MIP could introduce an additional systematic error. Jiang et al. [24] compared free breathing 3D-CT and AIP registrations to FB-CBCT and reported AIP to be more accurate than free breathing 3D-CT.

The second aim of this study was to evaluate differences between the automatic and manual registration for the same CT datasets. Comparing 3D shifts for the same CT datasets revealed the best agreement in median 3D shift for ∆AIP (1.1 mm) and also the smallest variability (1.1 mm). For some patients the differences in 3D couch shifts between automatic and manual registration were even larger than 5.0 mm which was suggested as internal target volume (ITV)-to-PTV margin to compensate for intra-fractional changes [31, 32]. Overall, the radiation oncologist assessed the tumor positions between the FB-CBCTs and the reference CTs somewhat different than the automatic registration. There is no comparison up to date on registration of different CT datasets with FB-CBCT and manual vs automatic registration, but some data are available on inter-observer variability. Sweeney et al. [22] compared registration results of three radiation oncologists and three radiotherapy technicians. They registered planning CTs to free breathing 3D-CBCTs or 4D-CBCTs and found an inter-observer variability for 3D-CBCT registration in SI direction of 1.5 mm (standard deviation) and 0.6 mm for 4D-CBCT. An inter-observer variability of the registration results could also be expected in our study, if manual registration would be performed by different observers.

The evaluation of correlations between tumor characteristics and registration differences showed, that 3D tumor motion affects the registration differences. This finding is in accordance to results from other groups [22, 24, 25]. Shift differences between the CT datasets as well as between automatic and manual registration increased with increasing 3D tumor motion. Manual registration was less affected by tumor motion than the automatic registration. SI distance showed correlations to registration difference between MIP and the other CT datasets too, which is due to the fact that SI distance of the tumors correlates also with tumor motion (*R*_s_ = 0.507, *p* < 0.01). In contrast to 3D tumor motion, tumor volume or tumor environment have only minor impact on registration results.

The AAPM task group 76 report [33] recommends the application of motion management for tumor motion greater than 5 mm. By dividing our patients into groups with tumor motion <5 mm and >5 mm, the automatic registration achieved significant differences between both groups for nearly all registrations, in contrast to manual registration. This in turn results in significant increasing 3D shift differences between automatic and manual registration with increasing 3D tumor motion, except for ∆MIP.

The larger the tumor motion the more different appears the tumor in the CT datasets. It is to be noted that asymmetrical breathing patterns can cause shifts in the depicted density distribution of the tumor. Such shifts were seen in FB-CBCT as well as in AIP but not in MIP, which can result in systematic positioning errors [25]. Furthermore, variations in the breathing patterns of patients can result in differences between CT acquisitions [34], which in turn impacts on the registration results. Such variations in breathing amplitude and frequency increase with increasing tumor motion.

In general, decreasing tumor motion would also decrease shift differences between different CT datasets and between automatic and manual registrations. Several techniques are available to decrease tumor motion amplitude (e.g. abdominal compression [35, 36]) or to restrict the irradiation to a certain breathing window (e.g. active breathing control [37, 38] or gating [39, 40]).

Compared to the other three reference CT datasets MidV CT is different in the tumor depiction. Originally, the MidV CT concept was dedicated to limit the margins for treatment planning [5]. MidV CT shows the tumor in a single phase during the breathing cycle in its time-weighted mean position. In comparison to FB-CBCT, the tumor depiction in MidV CT is sharper and not blurred (Fig. 1), which demands to use the ITV contour as assistance for the image registration.

Overall, the AIP seems to be the best option for image registration. Tumor depiction in AIP is similar to FB-CBCT and showed the best registration agreement as well for the automatic as for the manual registration. Therefore, AIP seems to be preferable to be used for treatment planning and image registration with FB-CBCT.

## Conclusion

The results of image registration with FB-CBCTs for patient positioning depend strongly on the applied reference CT dataset. Using PCT, AIP, MIP or MidV CT datasets for image registration with FB-CBCTs resulted in different couch shifts between the CT dataset as well as between automatic and manual registration. Median 3D shift differences for automatic registration were always smaller than for manual registration (0.9 mm on average). In more than 12 % of the patients registration differences between CT datasets larger than 5 mm were observed. Tumor motion has the largest impact on the registration results with larger registration differences for larger motion amplitudes. Except for ∆MIP, 3D shift differences for automatic vs manual registration were statistically significant if tumor motion was <5 mm and >5 mm. Shift differences between automatic vs manual registration were the smallest - with a median of 1.1 mm - for the AIP CT datasets due to similar tumor depiction in FB-CBCT and AIP. Therefore, AIP seems to be the most appropriate CT dataset for image registration with FB-CBCT.

## Abbreviations

4D-CBCT: Respiratory correlated CBCT
4DCT: Respiratory-correlated four-dimensional CT
AIP: Average intensity projection
COM: Center of mass
FB-CBCT: Free breathing cone beam CTs
GTV: Gross tumor volumes
IGRT: Image guided radiation therapy
ITV: Internal target volume
MidV: Mid-ventilation
MIP: Maximum intensity projection
OARs: Organs at risk
PCT: Slow planning CT
PTV: Planning target volume
R_S_: Spearman’s rank correlation coefficient
SBRT: Stereotactic body radiation therapy
SD: Standard deviation
SI: Superior-inferior
